# *ATP1A3*-related syndromes: our case-series unveiling a dynamic, fever-triggered and overlapping array of neurological phenotypes

**DOI:** 10.1007/s10072-026-09101-5

**Published:** 2026-05-19

**Authors:** G. Errichiello, P. Bernardo, F. Acquaviva, S. Troisi, M. Rosa, G. Bargiacchi, F. Esposito, A. Rubino, M. Carotenuto, A. Varone, L. D’Acunto

**Affiliations:** 1 Pediatric Neurology Unit, Department of Neurosciences, Santobono-Pausilipon Children’s Hospital, Naples, Italy; 2 Child Neuropsychiatry Unit, Department of Translational Medical Sciences, University of Federico II, Naples, Italy; 3 Child Neuropsychiatry Unit, Department of Neurosciences, Santobono-Pausilipon Children’s Hospital, Naples, Italy; 4 Medical Genetics Unit, Department of General and Emergency Paediatrics, Santobono-Pausilipon Children’s Hospital, Naples, Italy; 5 Pediatrics for Chronic and Multifactorial Diseases, Department of General and Emergency Pediatrics, Santobono-Pausilipon Children’s Hospital, Naples, Italy; 6 Child and Adolescent Neuropsychiatry, Department of Mental and Physical Health and Preventive Medicine, University of Campania “Luigi Vanvitelli”, Naples, Italy; 7 Institute of Neurology, Fondazione Policlinico Gemelli IRCCS, Rome, Italy

**Keywords:** *ATP1A3*, Pediatric movement disorder, FIPWE, RECA, CAPOS, Alternating hemiplegia of childhood (ACH), Flunarizine

## Abstract

**Introduction:**

ATP1A3-related neurological disorders show a broad spectrum of manifestations, usually with autosomal dominant transmission. Classical phenotypes include alternating hemiplegia of childhood (AHC), rapid-onset dystonia-parkinsonism (RDP), and syndrome characterized by cerebellar ataxia, areflexia, pes cavus, optic atrophy and sensorineural hearing loss (CAPOS). Additional rarer forms include childhood-onset-schizophrenia (COS), encephalopathy with MRI abnormalities without hemiplegia (D-DEMØ), fever-induced paroxysmal weakness and encephalopathy (FIPWE), and relapsing encephalopathy with cerebellar ataxia (RECA). These conditions often overlap, sharing core symptoms due to dysfunction of the Na⁺/K⁺-ATPase α3 subunit. Some mutations result in a thermolabile enzyme, which impairs its function under stress, leading to weakness’ episodes, encephalopathy and ataxia.

**Case series:**

We report a patients’ cohort with ATP1A3 mutations followed at Santobono-Pausilipon Children’s Hospital in Naples. The first family includes three siblings with RECA (p.Arg756Cys). The second cluster comprises a mother and son with FIPWE (p.Arg756His). We also describe one case of AHC (p.Asp801Asn) and one of CAPOS (p.Arg756Cys). All patients showed marked susceptibility to infection and fever.

**Discussion and conclusion:**

Our case series confirms the complex clinical scenarios in ATP1A3-related disorders, with symptoms overlapping and possible interfamilial variability, contributing to the diagnostic challenge posed by a rare genetic disorder, already observed in individuals with ATP1A3 gene mutations. The ongoing effort to characterize the clinical phenotype and identify “core” symptoms is necessary to expand our knowledge of the genotype-phenotype correlation, which is currently unclear. More importantly, our series highlights the molecular fragility of mutant ATP1A3, particularly its sensitivity to fever. Proactive prevention of fever and time management may be crucial to reducing the risk of neurological deterioration in affected individuals.

**Supplementary Information:**

The online version contains supplementary material available at 10.1007/s10072-026-09101-5.

## Highlights

**What are the main findings?:**

ATP1A3-related neurological disorders can be viewed as an umbrella term encompassing a spectrum of clinical conditions that often overlap in complex ways.

These disorders arise from mutations affecting the Na⁺/K⁺-ATPase α3 subunit, some of which render the enzyme distinctly thermolabile.

Thermal or metabolic stress can precipitate enzymatic dysfunction, triggering the signature paroxysmal episodes characteristic of this clinical spectrum.

**What is the implication of the main findings?:**

It would be highly advisable to implement hospital protocols aimed at preventing events as-sociated with thermal stress (such as vaccinations) in individuals at risk.

We present a series of cases characterized by significant intrafamilial variability observed in two multiplex families. This may contribute to expanding and refining the clinical spectrum of ATP1A3-related disorders.

**Supplementary Information:**

The online version contains supplementary material available at 10.1007/s10072-026-09101-5.

## Introduction

*ATP1A3*-related neurological disorders represent a heterogeneous spectrum of conditions with often overlapping clinical features and autosomal dominant inheritance [1].

The first case series, published in 1971, described eight children with paroxysmal hemiplegic episodes affecting one side of the body, with alternating laterality and accompanied by psychomotor developmental delay, dystonia or choreoathetosis [2].

In 1993, the first familial cluster syndrome with autosomal dominant expression was reported, characterized by rapid-onset dystonia parkinsonism (RDP) [3]. It took 19 years to identify the common cause of two seemingly unrelated conditions in the *ATP1A3* gene mutation [4]. In addition to the well-known clinical phenotypes of rapid-onset dystonia-parkinsonism (RDP) and alternating hemiplegia of childhood (AHC), another condition associated with missense mutation in the same gene was subsequently identified (1996). This condition, known as CAPOS syndrome, is characterized by cerebellar ataxia, areflexia, pes cavus, optic atrophy, and sensorineural hearing loss [5]. Initially, these three key phenotypes were thought to be distinct clinical entities. However, subsequent research has revealed a phenotypic continuum, with many patients presenting atypical or overlapping symptoms—such as cognitive or motor dysfunction, ataxia, hemiplegia, epilepsy, dysphagia/dysarthria, abnormal eye movements, and others. These features reflect a broad neurological spectrum, often with intermediate clinical presentations, particularly between AHC and RDP.

**Fig. 1:**
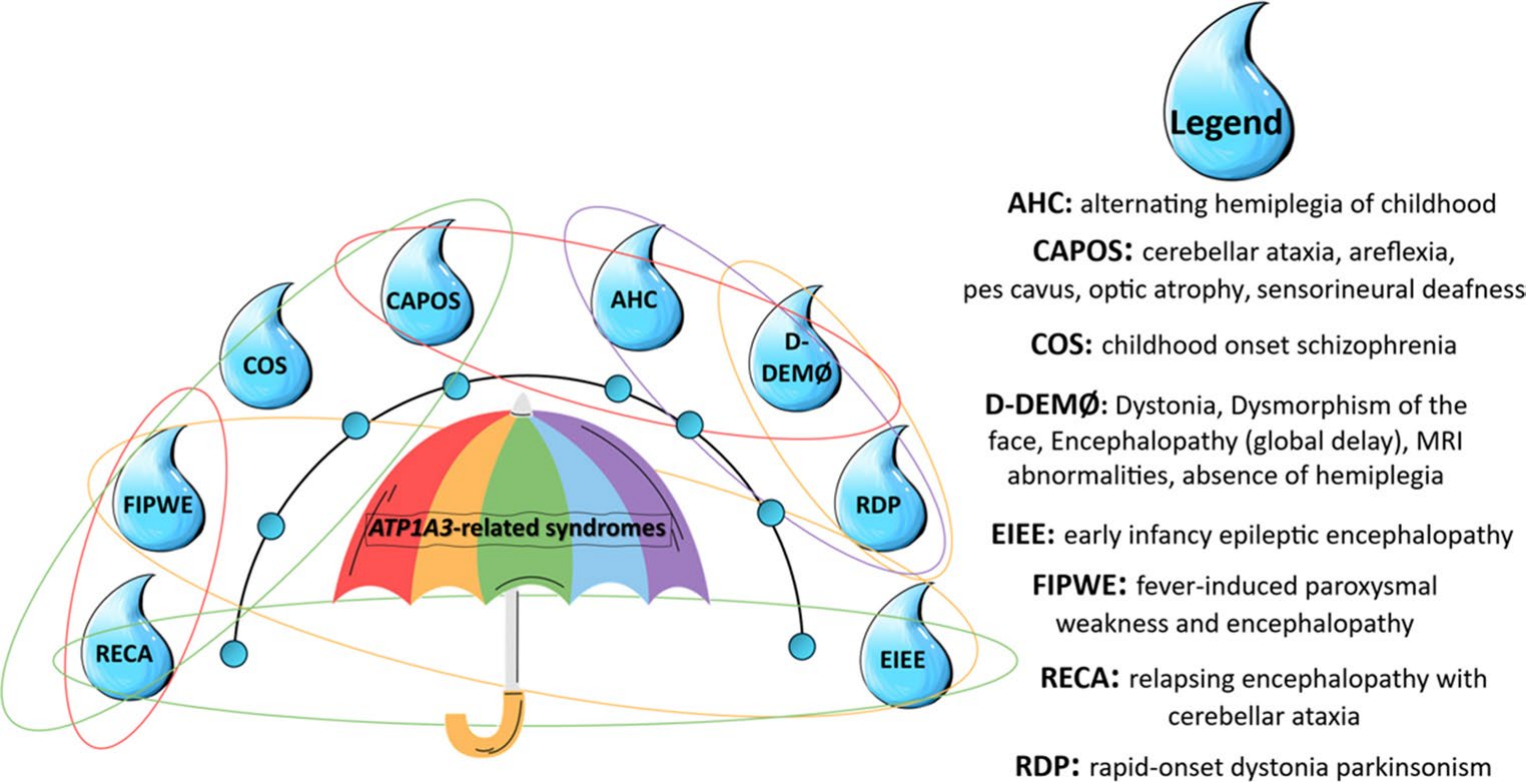
Graphical representation of ATP1A3-related disorder as an umbrella term including several conditions in phenotypic overlap with each other

Genotype-phenotype studies have identified additional conditions within the *ATP1A3*-related disorder spectrum, including:

- childhood-onset schizophrenia (COS),
- dystonia, facial dysmorphism, encephalopathy, MRI abnormalities, and absence of hemiplegia (D-DEMØ),
- fever-induced paroxysmal weakness and encephalopathy (FIPWE), and
- relapsing encephalopathy with cerebellar ataxia (RECA) [1] (see Fig. 1 and Table 1).

The *ATP1A3* gene encodes the α3 subunit of the Na⁺/K⁺-ATPase, a transmembrane protein crucial for maintaining neuronal function through ionic exchange, specifically, the transport of three sodium ions out of the cell in exchange for two potassium ions into the cell [6].

**Table 1:** Main clinical and genetic features’ ATP1A3-related neurological disorders

	Alternating hemiplegia of childhood (AHC)	Cerebellar ataxia, areflexia, pes cavus, optic atrophy, sensorineural deafness (CAPOS)	Childhood onset schizophrenia (COS)	Dystonia, Dysmorphism of the face, Encephalopathy, MRI abnormalities, absence of hemiplegia (D-DEMØ)	Fever-induced paroxysmal weakness and encephalopathy (FIPWE)	Relapsing encephalopathy with cerebellar ataxia (RECA)	Rapid onset dystonia parkinsonism (RDP)
Main clinical features	Episodic hemiplegia, dystonia, oculomotor abnormalities, seizures, cognitive delay	Acute ataxia, areflexia, sensorineural deafness, optic atrophy, pes cavus	Early onset psychosis, hallucinations, cognitive decline, movement abnormalities	Severe encephalopathy, microcephaly, deafness, ophthalmoplegia, refractory epilepsy	Acute flaccid weakness, encephalopathy, Seizures	Recurrent episodes of ataxia, dysarthria, encephalopathy	Sudden-onset dystonia, parkinsonism, bulbar symptoms
Age of onset	< 18 months	Early childhood	Late childhood/Adolescence	Neonatal	Infancy	Early childhood	Adolescence to adulthood
Brain MRI	Often normal or non-specific; sometimes cortical atrophy	Cerebellar atrophy in later stages	Cortical and subcortical atrophy, non-specific findings	Cerebral and cerebellar atrophy	Normal or mild/diffuse atrophy	Transient cerebellar swelling, cerebellar atrophy over time	Normal
Common ATP1A3 mutations	p.Asp801Asn (most common and milder phenotype), p.Glu815Lys (severe phenotype),	p.Glu818Lys, p.Arg756Cys/His	Rare variants (p.Gly316Ser, p. Leu924Pro)	De novo p.Gly316Ser	p.Gly316Ser, p.Ile274Asn, p.Arg756Cys/His	p. Asp801Asn, p.Arg756Cys/His	p.Thr613Met, p.Asp801Tyr

Physiologically, the Na⁺/K⁺-ATPase sustains the electrochemical membrane gradient and resting potential, regulates neuronal transmission and excitability, and restores ionic balance following synaptic activity. The α3 subunit is predominantly expressed in neurons with high energy demands, such as inhibitory GABAergic neurons, motor neurons, Purkinje cells, sensory neurons, and brainstem neurons. Accordingly, it is typically activated after high-frequency neuronal firing [7].

Recent findings indicate that *ATP1A3* deficiency not only impairs ion homeostasis but also compromises cellular stress responses and mitochondrial integrity, particularly under thermal stress. *ATP1A3*-deficient cells show reduced expression of the heat shock protein Hsp70, a key mediator of cell survival under stress, which correlates with impaired cell cycle progression, even at baseline. This intrinsic vulnerability is confirmed, rather than exacerbated, by heat exposure [8].

Mitochondrial dysfunction further contributes to this susceptibility. *ATP1A3*-deficient cells exhibit lower metabolic activity and reduced mitochondrial membrane potential, indicating compromised energy homeostasis that becomes critical during heat stress, when energetic demand increases [9].

These stress-related vulnerabilities may help explain the episodic nature of *ATP1A3*-related disorders, such as RECA and FIPWE, where fever or environmental stress can trigger encephalopathy, ataxia, or other fluctuating neurological symptoms [10].

## Materials and methods

We report a series of patients carrying *ATP1A3* mutations, all followed at the Santobono-Pausilipon Children’s Hospital in Naples during the last two years.

The first family includes three siblings diagnosed with RECA due to the p.Arg756Cys variant. The second involves a mother and her son with FIPWE associated with the p.Arg756His mutation. Additionally, we describe one patient with AHC (p.Asp801Asn) and another with CAPOS syndrome (p.Arg756Cys).

All patients presented with varying degrees of neurological and motor disability and were therefore treated as inpatients or day hospital patients. This was also to enable instrumental and genetic tests to be carried out, as well as clinical and pharmacological follow-up (Table 2).

**Table 2:** Main clinical and genetic features of our case history

	Family cluster 1	Family cluster 2	Case 3	Case 4
Main clinical features	Encephalopathy, hypotonia and psychomotor regression	Encephalopathy and progressive generalized dystonia	Seizures, alternating hemiplegic episodes, hypotonia, developmental delay	Psychomotor regression, hypotonia, craniofacial features,
Age onset	14 m.o (M) 2 y.o (M) 9 m.o (F)	4 y.o	3 m.o	At birth dismorphic features; 15 m.o psychomotor regression
Brain MRI	Negative	Negative	Negative	Supra- and subtentorial malformations
Paroxysmal events fever-triggered	Yes Psychomotor stagnation	Yes Dystonic postures	Yes/No Alternating hemiplegic episodes	Only at clinical onset
*ATP1A3* mutation	p.Arg756Cys	p.Arg756His	p.Asp801Asn	p.Arg756Cys
Diagnosis	RECA	FIPWE	AHC	CAPOS

### Genetic testing methods

All patients underwent next-generation sequencing (NGS) of genes associated with epileptic encephalopathies or movement disorders, except for one who underwent whole-exome sequencing (WES). The identified variants comply with the nomenclature of the American College of Medical Genetics and Genomics (ACMG) and the Association for Molecular Pathology (AMP).

## Case-series

### Family cluster 1

This cluster includes four children born to non-consanguineous parents. The mother had a history of para-infectious encephalopathy at age two. The first-born daughter had no clinical issues.

The second-born son developed axial hypotonia and psychomotor regression at 14 months, initially diagnosed as enteroviral encephalitis. Ataxia followed Measles, Mumps, Rubella, and Varicella (MMRV) vaccination.

The third child also exhibited psychomotor regression around age two, in a para-infectious encephalopathy context with generalized hypotonia. Laboratory and neuroimaging findings were inconclusive in both cases.

The youngest daughter had a similar onset, additionally experiencing a febrile seizure at nine months. The recurrence of symptoms raised suspicion of genetic etiology.

**Fig. 2:**
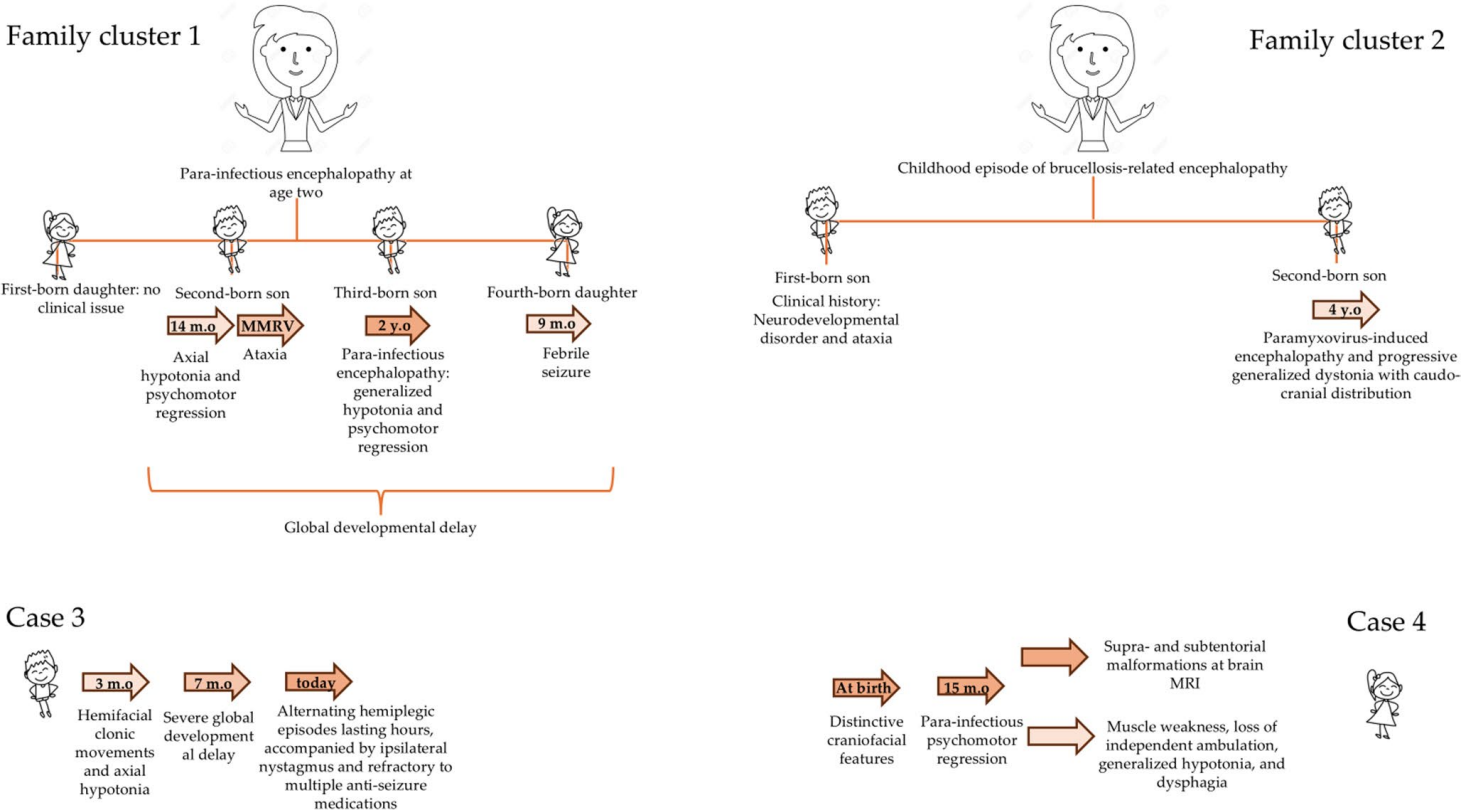
Graphical representation of patients’ clinical onset. *The brother of our patient, belonging to the second family cluster, has reached adulthood and is currently followed at another center; he was not included as we do not have sufficient data available

NGS for hereditary ataxia genes revealed a heterozygous pathogenic variant in *ATP1A3* (c.2266 C > T; p.Arg756Cys) in the third child, later confirmed in all affected siblings (Fig. 2).

### Family cluster 2

This cluster includes a five-year-old boy with Paramyxovirus-induced encephalopathy and progressive generalized dystonia with caudo-cranial distribution. The mother exhibited continuous dyskinetic movements and reported a childhood episode of brucellosis-related encephalopathy, never formally investigated. A family history of ataxia was noted; the boy’s brother is currently undergoing whole-exome sequencing (WES).

The child had normal development until age four, when, after an infection with fever and gastrointestinal symptoms, he developed gait disturbance. Initially, he showed generalized muscle weakness, diminished lower limb reflexes, and increased right-sided tone.

Neurological symptoms progressed to generalized dystonia, involving all limbs and oral-buccal muscles, impairing speech and swallowing (Video 1). Brain MRI, EEG, echocardiogram, and abdominal ultrasound were normal.

NGS targeting dystonia/dyskinetic syndrome genes identified two maternally inherited heterozygous variants:

- *VPS41*: c.1984 C > T (p.Arg662Ter), associated with autosomal recessive spinocerebellar ataxia in biallelic cases.
- *ATP1A3*: c.2267G > A (p.Arg756His), a pathogenic variant with autosomal dominant inheritance.

## Case 3

A boy presented at three months with a seizure characterized by gaze deviation and left hemifacial clonic movements, alongside axial hypotonia. EEG, echocardiogram, transfontanellar ultrasound, and fundus exam were normal.

Subsequent prolonged, contralateral seizures led to valproic acid therapy, later combined with carbamazepine with poor efficacy. Multiple EEGs remained normal.

Brain MRI showed mildly reduced posterior white matter and a millimetric SWI blooming artifact in the bulbar region, possibly a small cavernoma or hemosiderin deposit.

Neuropsychological assessment revealed severe global developmental delay at seven months: inability to sit, roll, crawl, or hold head in prone.

The clinical course included alternating hemiplegic episodes lasting hours, accompanied by ipsilateral nystagmus and refractory to multiple anti-seizure medications (carbamazepine, phenobarbital, nitrazepam, pyridoxine).

NGS for epileptic and developmental encephalopathies confirmed a de novo heterozygous pathogenic variant in *ATP1A3* (c.2401G > A; p.Asp801Asn), consistent with alternating hemiplegia of childhood.

## Case 4

The clinical history of the fourth case began at 15 months of age with para-infectious psychomotor regression, characterized by muscle weakness, loss of independent ambulation, generalized hypotonia, and dysphagia attributed to ineffective swallowing. At symptom onset, brain MRI revealed a complex pattern of supra- and subtentorial malformations, including anomalies of the cerebral commissures and pons, cerebellar hypodysplasia consistent with partial rhomboencephalosynapsis, and cortical developmental abnormalities suggestive of dysgyria.

The patient displays distinctive craniofacial features consistent with the spectrum of frontonasal dysplasia syndromes. These include macrocephaly, a broad forehead with frontal bossing and midline depression, hypertelorism, a partially bifid nasal tip, and an ogival palate. Additionally, the patient exhibits pectus excavatum and a prominent umbilical hernia.

Neuroimaging was extended to include an MRI of the entire spinal column, which was unremarkable.

To establish a definitive diagnosis, trio exome sequencing was performed, identifying two de novo heterozygous variants:

-*ATP1A3*: c.2266 C > T (p.Arg756Cys) and *EFNB1*: c.191G > T (p.Cys64Phe), both classified as pathogenic.

This final clinical case is even more complex. The brain malformations appear to be attributable to the *EFNB1* variant, whereas the craniofacial dysplasia is likely due to phenotypic overlap.

### Clinical follow-up

All patients are currently being monitored in day hospitals or specialist clinics.

They have all remained clinically stable, showing global psychomotor developmental delay or mild to moderate intellectual disability. Their neurological condition is also reasonably steady.

In the first family (RECA), psychomotor development plateaued in all three siblings. The three children have experienced paroxysmal movement disorder characterized by ataxia coinciding with fever relapses.

The second case (FIPWE) has been more difficult to manage, with recurrent post-febrile neurological deterioration and dystonia involving the hands, feet, and oral-buccal region. Treatment with trihexyphenidyl and tetrabenazine yielded partial benefits, resulting in a stable picture of generalized dystonia with limited relapses during fever. The addition of flunarizine was considered.

The third case (AHC) proved the most complex diagnostically and therapeutically, with alternating seizures and hemiplegic episodes. The patient was treated with various agents (valproic acid, carbamazepine, phenobarbital, pyridoxine, nitrazepam). After molecular diagnosis, topiramate was trialed for its carbonic anhydrase inhibition, with minimal effect. In contrast, flunarizine led to a marked reduction in episode frequency and duration, previously requiring frequent hospitalizations.

Finally, the multidisciplinary follow-up of the last clinical case includes checks to support all the associated symptoms of a stable clinical picture of global developmental delay.

## Discussion

The present case series describes the diagnostic challenge posed by a rare genetic disorder in a clinical scenario characterized by two families with variable intrafamilial phenotypic expression and two *de novo* onset cases with severe phenotype, extending the already described phenotypic heterogeneous observed in individuals with ATP1A3 gene mutations.

While the term “ATP1A3-related neurological disorders” included a broad evolving clinical spectrum, all our cases share key features: firstly, acute and progressive onset with febrile or para-infectious trigger; secondly, hyperkinetic movement disorder as a pre-dominant symptom, cognitive delay or stagnation, and limited diagnostic insight from neuroradiological or instrumental investigations.

A key emerging aspect of our cohort is the marked intrafamilial variability observed across the two multiplex families.

In the first family, three affected siblings and their mother, who previously experienced an encephalopathic episode but is currently well, carry the variant.

All three children show global developmental delay, whereas the affected mother illustrates substantially no symptoms in adulthood.

In the second family, both the mother and son are carriers. The mother presented with a late-onset (age 17) hyperkinetic syndrome characterized by generalized dystonia with encephalopathy and now she presents with non-disabling dyskinesias, whereas her child shows a significantly more disabling gait impairment documented on video (Video 1).

In both families, therefore, the parental phenotype is consistently milder than that of the affected offspring. As previously discussed, this likely reflects the impact of early disease onset: neurological insults occurring within the first year of life, during a critical developmental window, may disproportionately impair cognitive and motor trajectories.

Another peculiarity of our cohort is the coexistence of additional genetic findings in two patients. In the second family, the proband’s broader familial screening was negative, yet his affected brother carries a distinct ataxia-associated variant.

Similarly, case 4 harbors a second pathogenic mutation (*EFNB1* variant), with associated dysmorphic features and neuroradiological abnormalities that are likely attributable to the additional gene defect. The brain MRI abnormalities in case 4 are not clearly attributable to either of the two present mutations. The literature describes cases of brain abnormalities due to *EFNB1* mutations. For example, Ji Yoon Han et al. describe a case of a de novo mutation in a female infant with schizencephaly and dysgenesis of the corpus callosum [11], and several other cases of agenesis or dysgenesis of the corpus callosum are also reported [12]. In the context of CAPOS, however, specific alterations such as mild cerebellar atrophy are described [13]. Therefore, some elements are partially attributable to both mutations due to phenotypic overlap. The coexistence of diverse symptoms and multiple mutations underscores the complexity of these presentations and complicates the diagnostic process.

Across our series, the two de novo cases exhibited the most severe manifestations, particularly the third patient. Except for case 3, all individuals experienced post-infectious worsening, suggesting that para-infectious triggers play a central pathophysiological role.

From a genotype-phenotype perspective, particularly the relationship between thermolability and paroxysmal manifestations, the p.Arg756Cys variant is particularly heat-sensitive, leading to dysfunctional *ATP1A3* during febrile episodes. This disrupts protein folding, mitochondrial stability, and neuronal stress responses, contributing to acute neurologic deterioration [14].

The p.Arg756His variant also exhibits a degree of temperature-sensitive instability leading to rapid degradation and internalization of the α3 subunit at febrile temperatures (e.g., 39 °C), resulting in loss of pump activity. This is due to disruption of a stabilizing hydrogen bond network normally formed by Arg756 at the junction of membrane and cytoplasmic domains [15]. In both cases this sensitivity to temperature contributes to the paroxysmal neurological picture. The p.Asp801Asn variant accounts for 30–43% of cases of AHC [16]. Unlike p.Arg756His mutation, which has been shown to cause a true protein collapse at 39 °C, p.Asp801Asn variant is not structurally documented as thermolabile. However, it is well known that clinical episodes of AHC with mutations such as p.Asp801Asn can be precipitated by fever, infection or stress, probably due to an increase in functional neuronal demand in the presence of an already deficient pump. Clinically, this translates into fever-induced seizures, encephalopathy, motor regression, dystonia, or autonomic instability likely reflecting brainstem involvement and heat shock protein dysfunction [7].

Consequently, one of the key management strategies involves preventing fever and other infectious triggers that may precipitate relapses or worsen the clinical presentation because immunization may play a pivotal role in preventing infections that could act as fever-inducing triggers [17].

According to the LICE (Lega Italiana Contro l’Epilessia) guidelines, vaccinations should be performed without contraindication in children with epileptic encephalopathies (class of evidence III; strength of recommendation A) and concerns about epilepsy should not discourage immunization (class of evidence III; strength of recommendation A) [18]. Prophylactic antipyretics are recommended for patients with Dravet syndrome [19, 20]. Vaccination recommendations exist for developmental and epileptic encephalopathies, but evidence for ATP1A3-related disorders is still limited. For individuals with more severe phenotypes, structured hospital-based vaccination protocols with multidisciplinary assessment, may help clinicians both prevent and closely monitor potential risks.

## Conclusions

Our series underscores the significant intrafamilial variability and phenotypic complexity of ATP1A3-related disorders, shaped by early disease onset, para-infectious triggers, and, in some cases, the coexistence of additional genetic variants.

The contrast between milder parental phenotypes and more severe manifestations in progeny suggests critical vulnerabilities during early neurodevelopment.

The thermosensitive nature of *ATP1A3* variants also helps to explain the paroxysmal, fever-induced deterioration observed in cases. These findings emphasize the importance of preventive strategies, including hospital-based vaccination protocols involving multidisciplinary assessments and fever management.

However, they also highlight the broader challenge of identifying the various contributing factors within an expanding ATP1A3 clinical spectrum.

## Electronic Supplementary Material

Below is the link to the electronic supplementary material.

Supplementary Material 1 (MP4 1.44 MB)

Supplementary Material 2 (MP4 143 KB)

Supplementary Material 3 (PDF 682 KB)

## Data Availability

No datasets were generated or analyzed for this case series. All information presented in the manuscript is contained within the article.
